# Relationship between evaluation factors and star ratings for Japanese community healthcare institutions in electronic word-of-mouth reviews: an observational study

**DOI:** 10.1186/s12875-024-02668-y

**Published:** 2024-12-05

**Authors:** Hiroki Maita, Yuki Kanezaki, Takashi Akimoto, Tadashi Kobayashi, Takahiro Hirano, Hiroyuki Kato

**Affiliations:** 1 Development of Community Healthcare, Hirosaki University Graduate School of Medicine, 5 Zaifu-cho, Hirosaki-shi, Aomori 036-8562 Japan; 2 Hirosaki University School of Medicine, 5 Zaifu-cho, Hirosaki-shi, Aomori 036-8562 Japan; 3 Department of General Medicine, Hirosaki University School of Medicine & Hospital, 53 Hon-cho, Hirosaki-shi, Aomori 036-8563 Japan; 4 General Medicine, Hirosaki University Graduate School of Medicine, 5 Zaifu-cho, Hirosaki-shi, Aomori 036-8562 Japan

**Keywords:** EWOM, Healthcare institution, Internet review, Star rating, Word-of-mouth

## Abstract

**Background:**

Internet reviews have become increasingly crucial for both healthcare providers and patients. Electronic word-of-mouth (eWOM) reviews on internet sites often comprise textual content with numerical ratings. In this study, we aimed to identify the evaluation factors of community healthcare institutions regarding eWOM reviews and the impact of each evaluation factor on the institution’s ratings.

**Methods:**

An observational study was conducted to qualitatively and quantitatively analyse eWOM data posted on Google for randomly selected healthcare institutions in Hirosaki, Japan from September to October 2022. For qualitative data, the authors repeatedly read the eWOM text, coded it, and categorised related sections. For quantitative analysis, a multivariate analysis using a linear regression model was conducted with the categorised factors from the qualitative analysis as explanatory variables and eWOM ratings as response variables.

**Results:**

Twenty medical institutions (two hospitals and 18 clinics) were randomly extracted from the registry. A total of 147 eWOM texts from each institution were analysed, and coding was performed for 474 segments in the texts. In the qualitative analysis, the evaluated factors in eWOM texts for medical institutions were categorised as communication (evaluation factor for communication with healthcare providers), clinical practice (evaluation factor for the clinical practice of healthcare providers), and medical institution (evaluation factor for the characteristics of medical institutions). According to the multiple regression analysis, the partial regression coefficients for the explanatory variables of positive communication, clinical practice, and medical institution evaluations were 1.04 (95% confidence interval 0.70 to 1.39), 0.65 (95% confidence interval 0.27 to 1.04), and 0.75 (95% confidence interval 0.35 to 1.15), respectively, with the number of ratings as the response variable. Partial regression coefficients for the explanatory variables of negative communication, clinical practice, and medical institution evaluations were − 1.52 (95% confidence interval − 1.88 to -1.16), -0.90 (95% confidence interval − 1.28 to -0.52), and − 0.24 (95% confidence interval − 0.60 to 0.12), respectively.

**Conclusion:**

We quantitatively and qualitatively analysed the eWOM reviews and ratings of healthcare institutions posted on Google. Three evaluation factors were identified: communication, clinical practice, and medical institution. Our study revealed that communication significantly impacts ratings.

**Supplementary Information:**

The online version contains supplementary material available at 10.1186/s12875-024-02668-y.

## Background

The Internet has led to significant changes in the landscape of information available to consumers. According to Japan’s Ministry of Internal Affairs and Communications’ White Paper on Information and Communications, internet usage rates in Japan have been steadily increasing, standing at 82.9% in 2021 [1]. A survey conducted by KDDI Evolva, a provider of business process outsourcing services in Japan, revealed that 94.0% of consumers gather pre-purchase information when buying products or services, and 50.6% indicated that word of mouth influences their purchase decisions [2]. In the field of healthcare, electronic word-of-mouth (eWOM) information, including reviews on platforms such as Google, is being increasingly utilised as a criterion for selecting physicians; its importance has steadily increased in recent years [3]. According to a survey conducted by M3, Inc., a medical portal site service provider in Japan, 68.3% of the respondents reported using some form of word-of-mouth information when choosing a healthcare facility. While recommendations from family and acquaintances accounted for a significant proportion (19.1% and 16.6%, respectively), the use of eWOM has become prevalent particularly among the younger generation, with a reported rate of 26.4% among individuals in their 20s (compared with 7.9% across all age groups) [4].

According to a study outside Japan that analysed word-of-mouth factors through a questionnaire survey, four factors (communication skills, expertise, reputation and success, and institutional facilities) directly and indirectly influence patient satisfaction [5]. Recent systematic reviews reveal that physician rating websites, while potentially useful for supporting decisions based on patient perceptions and for market research, provide limited insights into the actual medical quality of physicians [6]. Numerous studies outside healthcare have examined eWOM to identify factors influencing the evaluation of products and services [7–9]. However, in the healthcare field, limited domains (such as cosmetic surgery [10], online gynaecological communities [11], specialised drug treatment facilities [12], and a single regional teaching hospital [13]) have been examined. Although some studies have classified eWOM for Japanese healthcare institutions according to previously identified evaluation factors [14], research that qualitatively elucidates the evaluation factors themselves from eWOM text data is lacking.

eWOM reviews often contain textual content and numerical ratings, such as star ratings. The rating is merely a numerical value, and the results of the evaluation can only be inferred from the text. Therefore, we would expect an indirect improvement in the rating by intervening in the factors indicated in the text. For this purpose, we must identify the evaluation factors and analyse their impact on ratings. Several studies have reported an association between ratings and evaluation factors. One study was conducted on specialised drug treatment facilities in the United States [12], and another study evaluated medical facilities in a prefecture in Japan [14]. The former reported a high correlation of a focus on recovery and the helpfulness of staff with high ratings, while the latter reported that doctors’ behaviour impacts high ratings. Both studies focused on medical facilities with either 5-star or 1-star ratings. Unlike our study, no previous study directly relates the continuous numerical values of ratings to the categories extracted from the text. Healthcare providers can improve patient satisfaction by effectively utilising the evaluation factors derived from posted eWOM. This benefits providers and patients, as satisfaction levels can be shared and thus anticipated when seeking healthcare services.

Therefore, we aim to qualitatively elucidate the evaluation factors of healthcare institutions and quantitatively determine how they influence the overall star ratings using eWOM information about Japanese healthcare institutions posted on Google.

## Methods

As this study did not involve human subjects and dealt only with public information on the Internet, the Ethics Committee of Hirosaki University waived the requirement for institutional review board approval.

There are a total of 177 medical institutions (14 hospitals and 163 clinics) in Hirosaki City listed in two medical institution registers (Aomori Prefecture Health and Welfare Related Institution Register and Medical Institution Register), available on the Aomori Prefectural Government’s website. The medical institutions were serialised, and the final sample was selected by generating random numbers on a computer to avoid selection bias and ensure representativeness. We adopted an exploratory sequential design, a type of mixed methods study [15]. The process begins with qualitative data collection and analysis, followed by quantitative data collection and analysis, and finally, interpretation.

From September to October 2022, a qualitative analysis of Google eWOM texts for each extracted healthcare institution was conducted, and its relationship with ratings (on a 5-point scale) was analysed. The inclusion criterion for incorporating eWOM data was that the data should contain extractable text and ratings. Exclusion criteria included (1) obvious misstatements (e.g. comments on another facility) and (2) unclear evaluation perspective and inability to analyse factors (e.g. extremely short evaluation comments such as ‘good’ or ‘awful’). For the qualitative data, the authors repeatedly read the eWOM text and coded and categorised the sections in the text as evaluation factors for medical institutions using NVivo (NVivo Release 1.5, QSR International Inc., U.S.). For example, if a reviewer commented, ‘The apology was followed by excuses and attempts to shift blame’, the patient may evaluate the doctor’s attitude towards the apology. Therefore, in this case, we classified the category as ‘attitude’ and the subcategory as the attitude towards the ‘apology’. The number and content of comments varied among medical institutions, making it theoretically challenging to estimate the sample size required to achieve theoretical saturation in qualitative analysis or calculate the sample size needed for quantitative analysis (such as multiple regression analysis). Initially, we conducted a qualitative analysis of the eWOM of the first 20 randomly selected medical institutions. One of the most common biases in qualitative analysis is confirmation bias, which occurs when a researcher interprets data in a way that favours their hypothesis. To minimise this risk, we implemented a rigorous multi-step analysis process. Initially, HM, YK, and TA conducted open coding for all 20 sampled medical institutions, using inductive reasoning to identify categories within the derived themes. In cases of disagreement, they engaged in thorough discussions to reach a consensus. Subsequently, TK, TH, and HK validated the data and reviewed the coding to ensure accuracy and consistency, followed by a team-wide discussion to finalise the coding and ensure comprehensive agreement. Throughout this process, we thoroughly reviewed all obtained data with multiple researchers and repeatedly analysed them to ensure a clear and less biased interpretation. If theoretical saturation was not achieved in the initial qualitative analysis, we scheduled additional sampling, known as theoretical sampling, which is driven by the need to collect more data to examine categories further and ensure representativeness within the categories [16]. We based our categorisation approach on Herzberg’s two-factor (motivation–hygiene) theory and Kano’s three-factor theory of customer satisfaction, which states that positive and negative rating scales are not continuous [17]. Therefore, we conducted a qualitative classification of positive and negative evaluations independently of the star ratings to facilitate subsequent quantitative analysis. We defined positive evaluations as comments expressing satisfaction, praise, or favourable experiences, while negative evaluations were those expressing dissatisfaction, criticism, or unfavourable experiences. EZR (EZR ver. 1.54, Saitama Medical Center, Jichi Medical University, Japan), a modified version of R Commander designed to add statistical functions and frequently used in biostatistics, was used to analyse the quantitative data [18]. Multivariate analysis using a linear regression model was conducted with categorised evaluation factors as explanatory variables (binary variable with presence or absence) and star rating numbers as response variables. The partial regression coefficient indicates the extent to which the presence of a rating comment in any category raises or lowers the star rating. Although random errors must be considered in quantitative studies, we adopted a strategy to evaluate the significance of the partial regression coefficients in the final multiple regression equation, providing statistical validity to this study’s results.

## Results

Twenty medical institutions (two hospitals and 18 clinics) were randomly extracted from the medical institution registry. Further, a total of 147 eWOM texts for the selected institutions were analysed, and coding was performed for the 474 segments in the texts.

In the qualitative analysis, the factors evaluated by eWOM for medical institutions were grouped into three categories: communication (evaluation factor for communication with healthcare providers), clinical practice (evaluation factor for the clinical practice of healthcare providers), and medical institution (evaluation factor for the characteristics of medical institutions). Each category consisted of several subcategories. The communication category comprised three subcategories: attitude, content of statements, and appearance. The clinical practice category comprised eight subcategories: medical examination, test, diagnosis, explanation, treatment, medical coordination, general practice, and risk management. Finally, the medical institution category comprised four subcategories: facility, system, staff, and healthcare delivery level (Table 1). The structures of the subcategories and the codes for each category are listed in Table S1a–c.

**Table 1:** Classification of the evaluation factors for word-of-mouth text (*n* = 474)

Categories (*n*)	Subcategories (*n*)	Evaluation (*n*)
Communication (226)	Attitude (213)	P (130)
		N (83)
	Content of statements (11)	P (0)
		N (11)
	Appearance (2)	P (2)
		N (0)
Clinical practice (152)	General practice (46)	P (31)
		N (15)
	Treatment (33)	P (13)
		N (20)
	Explanation (29)	P (15)
		N (14)
	Diagnosis (18)	P (11)
		N (7)
	Medical coordination (10)	P (1)
		N (9)
	Interview and physical examination (9)	P (7)
		N (2)
	Risk management (5)	P (0)
		N (5)
	Test (2)	P (1)
		N (1)
Medical institution (96)	System ss(45)	P (10)
		N (35)
	Facility (43)	P (28)
		N (15)
	Healthcare delivery level (5)	P (4)
		N (1)
	Staff (3)	P (1)
		N (2)

Note. *n* number of segments in word-of-mouth text, *N* negative evaluation, *P* positive evaluation

In the quantitative analysis, the highest number of textual segments was related to the communication category (*n* = 226), followed by clinical practice (*n* = 152) and medical institution (*n* = 96). In the communication category, the attitude subcategory accounted for the most segments (213/226) (Table 1). Among all textual segments, there were 254 positive evaluation segments and 220 negative ones. According to the multiple regression analysis, the partial regression coefficients for the explanatory variables of positive communication, clinical practice, and medical institution evaluations were 1.04 (95% confidence interval [CI] 0.70 to 1.39), 0.65 (95% CI 0.27 to 1.04), and 0.75 (95% CI 0.35 to 1.15), respectively, with the number of ratings as the response variable. Partial regression coefficients for the explanatory variables of negative communication, clinical practice, and medical institution evaluations were − 1.52 (95% CI −1.88 to −1.16), −0.90 (95% CI −1.28 to −0.52), and − 0.24 (95% CI −0.60 to 0.12), respectively. The coefficient of determination was 0.69 (Table 2).

**Table 2:** Impact of word-of-mouth textual factors on ratings evaluated with multiple regression analysis

Categories	Evaluation	Partial regression coefficient (95% confidence interval)	*p*-value
Communication	P	1.04 (0.70 to 1.39)	<0.01
	N	−1.52 (−1.88 to −1.16)	<0.01
Clinical practice	P	0.65 ( 0.27 to 1.04)	<0.01
	N	−0.90 (−1.28 to −0.52)	<0.01
Medical institution	P	0.75 ( 0.35 to 1.15)	<0.01
	N	−0.24 (−0.60 to 0.12)	0.2

Note. *n* number of segments in word-of-mouth text, *N* negative evaluation, *P* positive evaluation

## Discussion

We identified three categories of rating factors for eWOM—communication, clinical practice, and medical institution—and 15 subcategories, mentioned above. The number of eWOMs was highest in the communication category (particularly regarding the attitude subcategory), which also had the most significant impact on the number of ratings.

### Evaluation factors for medical institutions by eWOM

In this study, communication was treated as related to everyday life and not as a professional skill. In contrast, the category of clinical practice was treated as a concept that included the medical professionalism of healthcare providers. While the categories of communication and clinical practice were intended for people such as doctors or nurses, the medical institution category was treated as an item intended for hospitals or clinics. In the categorisation process, we attempted to incorporate perspectives from authors with different backgrounds (multiple physicians and medical students), referring to previous reports [5, 7–11, 13, 14, 19] to create rigorous and reliable categories consistent as evaluation criteria [20].

In general, eWOM readers are interested in the actual content of a review and not just its rating. Factual reviews and reviews with narrative or experiential styles are perceived as more beneficial [21]. In addition to the review style, the factors evaluated are also crucial. In an analysis of word-of-mouth reviews outside of Japan related to physician selection, four factors were reported to directly and indirectly influence patient satisfaction: communication skills, expertise, reputation and success, and institutional facilities [5]. Another report observed the quality of healthcare services, their price, and personal and contextual factors related to the patient as major determinants of patient satisfaction [22]. Empathy was also reported to be a service quality element that directly influences word of mouth [23]. Another report stated that outcome quality is the most important factor influencing patient dissatisfaction, followed by administrative, interactive, and environmental qualities [24]. However, the results of a Japanese patient survey indicated that communication was the most important factor affecting overall satisfaction [25]. The process of categorisation is inevitably influenced not only by the research subjects but also by the individual interests of each researcher. However, the three categories and 15 subcategories (Table 1 and S1a–c) presented in this study, while differing in perspectives and hierarchical differences in categorisation, are consistent with the findings of previous studies.

### Rating prediction using text data

eWOM reviews often contain text and quantitative ratings, such as star ratings. In general, text and ratings are considered to be related; however, their respective impacts on readers do not necessarily coincide [26]. For online purchasing platforms, negatively valenced reviews tend to supersede star ratings; when a review is positive, consumers trust the star ranking system, perceiving a high star ranking as the most credible [27]. Word-of-mouth communication influences the purchase of products and services. Negative word-of-mouth messages have a greater impact than positive ones [11, 28], and dissatisfied patients tend to be more active in negative postings [24]. Therefore, in healthcare institution evaluations, it is essential to take an approach that improves both eWOM rating scores and the content of text reviews. Addressing the evaluations of dissatisfied posters, in particular, can become a crucial challenge for healthcare providers. For healthcare providers to approach this efficiently, quantifying the relationship between evaluation factors in the text and ratings is essential. Specifically, numerically demonstrating the impact magnitude of each evaluation factor on ratings can help implement practical initiatives with clear priorities.

To analyse the relationship between ratings and evaluation factors, we used a linear regression model to create a rating prediction equation and compared the partial regression coefficients for each evaluation factor to quantitatively estimate their impact on the ratings (Table 2). The submitted review texts contained extensive communication-related content, which had a significant relationship with the ratings. Specifically, the effect on ratings was greatest when the text contained negative communication factors, with a decrease of 1.51 on a 5-point rating scale. This study’s results align with previous reports [10, 23], that negative factors have a more significant impact than positive factors.

### Role and prospects of eWOM in healthcare services

The three categories and 15 subcategories identified in our analysis provide a framework for systematic improvement. Building on our finding that communication factors significantly impact ratings, healthcare providers should prioritise addressing communication issues, particularly negative ones, to improve their eWOM ratings. With the expansion of communication through the Internet and intensified competition among companies, eWOM marketing strategies have become crucial for organisations [29, 30], even in the healthcare sector [31]. Treatment outcomes are suggested to serve as essential evaluation indicators; however, the absence of efforts to attract potential patients through positive word of mouth can also impact the sustainability of healthcare institutions [25]. This is evidenced by recent events; in April 2024, a group of Japanese doctors filed a civil lawsuit against Google, seeking damages for defamatory and often false comments.

Health information influences patients’ health behaviours; however, true freedom in selecting healthcare institutions is only possible based on high-quality and sufficient medical information. Moreover, owing to the difficulty in accurately evaluating healthcare services from patients’ perspectives, the demand for simplified healthcare information that patients can comprehend is increasing [32]. One way to respond to this is to utilise eWOM, which influences consumers, especially in the digital healthcare field, where information asymmetry is prominent [33]. However, the quality of health information on the Internet, including eWOM, is generally suboptimal and often lacks content that the public can reliably trust [34]. Our categorisation of evaluation factors could also serve as a framework for organising and presenting such information.

Although eWOM represents subjective evaluations from contributors and may lack accuracy and reliability, reports have suggested a correlation between rating systems and actual clinical outcomes for patients [19, 35, 36]. In the context of healthcare decision-making, patients perceive online information as a supplementary resource rather than a challenge. Consequently, healthcare providers should effectively utilise this information to improve their services [37]. By focusing on the categories identified in this study, particularly communication factors, and addressing both positive and negative reviews, healthcare providers can work towards improved healthcare quality, patient satisfaction, and potentially, better clinical outcomes.

### Limitations

This study has several limitations. First, eWOM spreads not only through Google but also through various social media platforms such as Facebook, Instagram, YouTube, and Twitter. Different information sources and services, as well as the presence of visual information such as photos, may lead to varying results [38]. Further studies must investigate these factors. Second, individuals posting these reviews remained anonymous, preventing the analysis of personal information, such as age and gender. The results are limited to individuals who can post online reviews; hence, caution is needed when generalising the findings. A detailed analysis is only possible with access to limited information for research purposes. Third, there is a likelihood of sponsor bias, a subset of contributor bias. Such bias may arise from scenarios where institutional representatives encourage employees to submit positive evaluations or when professional rivals deliberately seek to undermine the credibility of other entities. Given that Google ratings are anonymous and lack mandatory conflict of interest declarations, it becomes impracticable to definitively exclude the presence of these biases. Fourth, we employed multiple regression analysis to examine the impact of various attributes on overall customer satisfaction. While this approach provided insights relevant to our dataset, it did not fully align with Kano’s three-factor model [17] of customer satisfaction, which suggests an asymmetric relationship between attribute-level performance and overall satisfaction. Using multiple regression may oversimplify the nuanced impacts of basic, one-dimensional, and delighting factors within this framework. To better capture these asymmetric effects, future research could utilise alternative analysis methods such as penalty–reward–contrast analysis or nonlinear equation modelling, which have been suggested as viable options for analysing attribute impact under the three-factor structure [39–41]. Additionally, previous studies that attempted to predict ratings using review texts from e-commerce websites have shown that neural network regression models are more accurate than linear regression models [26]. Ratings can vary significantly among individuals and may not necessarily align with the content of the review text (review bias). Therefore, verification through studies using alternative evaluation methods is required. These methods may provide more precise insights into how each attribute influences customer satisfaction within the context of Kano’s model. Finally, in Japan, where this study was conducted, patients have the freedom to choose the medical institutions they visit, especially in the case of primary care facilities. The universal health insurance system alleviates the financial burden on patients, ensuring that the cost of medical treatment remains consistent across medical institutions. Consequently, patients place significant importance on word of mouth and other forms of information when selecting a medical institution. Given the fewer restrictions on medical visits and the reduced financial burdens, evaluations may focus on the quality of medical services at each facility, which is a strength of this study. However, ratings and the content of online reviews may vary depending on the healthcare resources and cultural backgrounds (countries or regions) underlying the study. Therefore, caution should be exercised when generalising these findings.

## Conclusions

We quantitatively and qualitatively analysed eWOM reviews and ratings related to healthcare institutions, identifying three categories and 15 subcategories as evaluation factors. Among these, the communication category was found to significantly impact overall ratings. In our increasingly information-driven society, such word-of-mouth data have become a valuable resource for both healthcare providers and patients. Analysing and leveraging eWOM is crucial for enhancing healthcare service quality, as it significantly impacts both the sustainability of healthcare institutions and patient decision-making processes. By systematically examining patient reviews through the framework developed in this study, healthcare providers can identify specific areas for improvement in their services, potentially leading to increased patient satisfaction and better clinical outcomes. Future research should explore the effects of eWOM across various social media platforms beyond Google reviews to provide a more comprehensive understanding of patient perspectives. Additionally, developing robust methods to address issues of anonymity and potential bias in online reviews would enhance the reliability and applicability of eWOM analysis in healthcare settings.

## Electronic supplementary material

Supplementary Material 1

## Data Availability

The data analysed in this study are included in this published article. Additional data are available from open sources on the Internet.

## Abbreviations

CI: Confidence interval
eWOM: electronic word-of-mouth
